# Long-Term Stability and Efficacy of NCT Solutions

**DOI:** 10.3390/ijms25168745

**Published:** 2024-08-10

**Authors:** Gabriel J. Staudinger, Zach M. Thomas, Sarah E. Hooper, Jeffrey F. Williams, Lori I. Robins

**Affiliations:** 1 Physical Sciences Division, University of Washington Bothell, Bothell, WA 98011, USA; gjstauds@uw.edu (G.J.S.); zachmt@uw.edu (Z.M.T.); 2 Microbiology & Infection Research Group, Cardiff School of Sport and Health Sciences, Cardiff Metropolitan University, Cardiff CF5 2YB, UK; smaddocks@cardiffmet.ac.uk; 3 Briotech Inc., 1102 Shuksan Way, Everett, WA 98203, USA

**Keywords:** *N*-chlorotaurine, antimicrobial, anti-inflammatory, wounds

## Abstract

To realize the potential for the use of *N*-chlorotaurine (NCT) in healthcare, a better understanding of the long-term stability of the compound in water is needed. An array of analytical procedures is required that can measure changes in NCT concentration over time and allow for the detection and identification of contaminants and likely degradation end products. We used UV-Vis and NMR spectroscopy, HPLC, and LCMS to establish the stability of NCT in solutions subjected to prolonged ambient and elevated temperatures. Stability proved to be dependent on concentration with half-lives of ~120 days and ~236 days for 1% and 0.5% solutions of NCT at ~20 °C. Regardless of initial pH, all solutions shifted toward and maintained a pH of ~8.3 at 20 °C and 40 °C. NCT at 500 µg/mL and 250 µg /mL inhibited biofilm formation by *Pseudomonas aeruginosa* and *Staphylococcus aureus* but did not disperse established biofilms. NCT exposure to the biofilms had profound effects on the viability of both bacteria, reducing live organisms by >90%. Exposure of Interleukin-6 (IL-6) to 11 µM NCT reduced the binding of IL-6 to an immobilized specific antibody by ~48%, which is 5× the amount required for HOCl to bring about the same effect in this test system. Our data demonstrate the potency of the compound as an antimicrobial agent with potential benefits in the management of infected chronic wounds and suggest that NCT may contribute to anti-inflammatory processes in vivo by direct modification of cytokine mediators.

## 1. Introduction

Chlorination of intracellular taurine follows the myeloperoxidase generation of hypochlorous acid and results in the formation of *N*-chlorotaurine (NCT) [1]. The enduring broad-spectrum antimicrobial properties of NCT and its now well-characterized interactions with inflammatory mediators suggest a central role for the compound in resolution and restoration events that arise after injury and infection in vivo [2,3,4,5]. The properties support the likelihood that exogenously supplied sources of synthetic NCT would prove therapeutically beneficial in a wide range of disease conditions, exemplified by published reports of its effectiveness in combating bacterial, viral, fungal, and protozoal infections [2,3,6,7].

The development of NCT for these purposes requires the application of rigorous analytical characterization methods to satisfy regulatory expectations of purity and stability. An equally thorough understanding of the dose–response relationships will determine the likely ranges of biological efficacies for various interventions. Here, we describe the application of spectrophotometric, chromatographic, and spectroscopic analytical methods to the characterization of aqueous solutions of laboratory-synthesized NCT after storage under a variety of conditions. We also report on the antimicrobial efficacy of NCT against *Pseudomonas aeruginosa* and *Staphylococcus aureus* and the impact of the compound on the immunogenicity of IL-6 cytokine in an antibody-binding assay. 

## 2. Results

**Figure 1:**
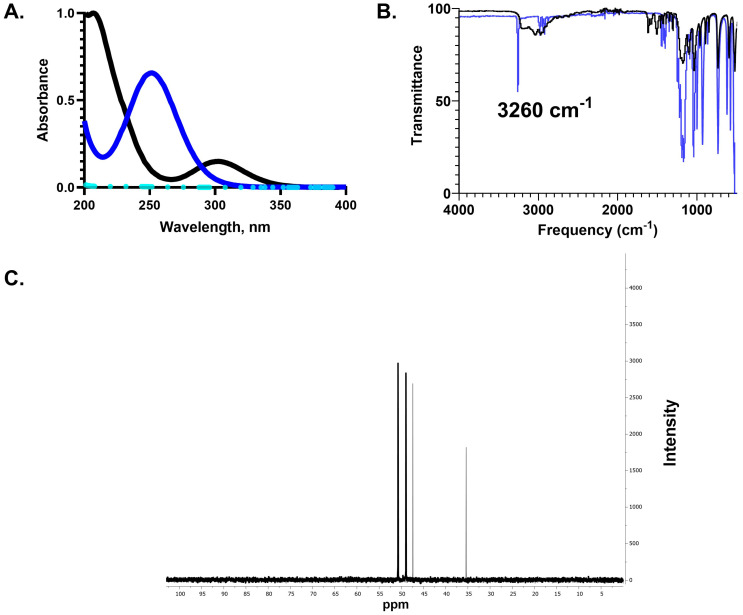
UV-Vis spectroscopy, IR spectroscopy, and ^13^C NMR spectroscopy of NCT. (**A**) UV–Visible spectroscopy wavelength scans of NCT (blue), N,N-dichlorotaurine (black), and taurine (teal). (**B**) IR spectrum of taurine (black) and NCT (blue). (**C**) ^13^C NMR spectrum of NCT (black, singlets at 50.74 and 48.96 ppm) and taurine (gray, singlets at 47.42 and 35.38 ppm).

**Figure 2:**
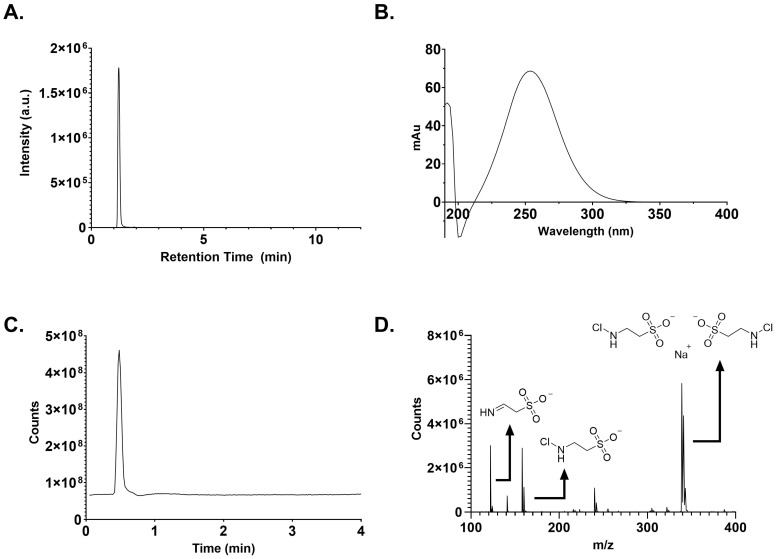
HPLC and LCMS analysis of NCT. (**A**) HPLC spectrum of NCT (1 mg/mL) at 252 nm. The area under the curve was calculated and purity was determined to be >95%. (**B**) UV-Vis wavelength trace of NCT detected by the LCMS. (**C**) TIC data for the corresponding LCMS peak at 252 nm. (**D**) MS data for the corresponding TIC peak at 252 nm.

NCT was characterized by UV–Visible spectroscopy as seen in Figure 1. As expected, the λ_max_ for NCT is at 252 nm [3]. N,N-dichlorotaurine, generated by adding 0.1 M HCl to NCT, showed a shift in λ_max_ to 306 nm. Taurine showed no absorbance at the measured wavelengths. IR spectroscopy showed the expected NH stretch for NCT at 3260 cm^−1^ and ^13^C NMR spectroscopy confirmed the expected difference between chemical shifts (Figure 1) [3]. LCMS was used to further characterize NCT (Figure 2).

As seen in Figure 2, a single peak at 252 nm by HPLC was detected with >95% purity. Starting synthetic reagents and N,N-dichlorotaurine were not detected. LCMS analysis showed the expected UV peak at 252 nm and verified purity >95%. The Total Ion Chromatogram (TIC) for the corresponding peak at 252 nm showed a single peak. The extracted masses from the TIC included NCT (157.9684 *m*/*z*), a sodium ion dimer of NCT (338.9261 *m*/*z*), and the elimination imine product (121.9915 *m*/*z*) (Figure 2). An additional TIC peak seen at a later acquisition time, likely a by-product of the instrument, with a *m*/*z* ratio of 187.8978, was detected. This may be an unsaturated form of N,N-dichlorotaurine [C_2_Cl_2_NO_3_S]^−^, which represents less than 4% of the relative ion count of NCT. 

**Figure 3:**
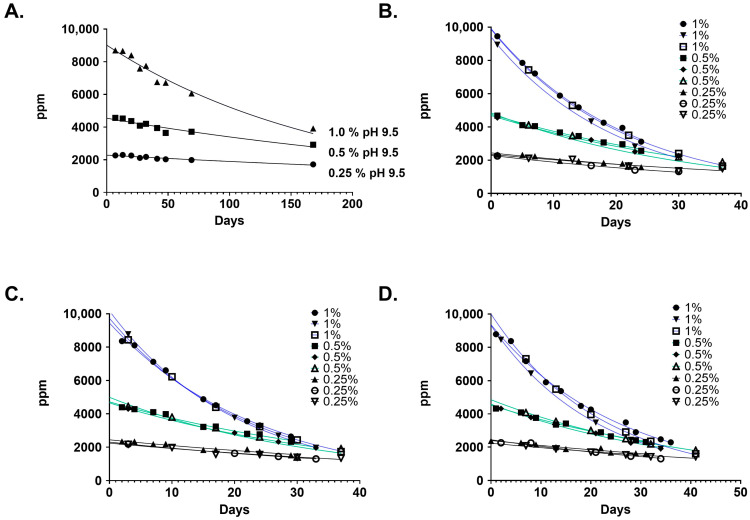
Stability of aqueous NCT solutions at 1%, 0.5%, and 0.25% at ambient and elevated temperatures. (**A**) Starting pH 9.5 at room temperature. (**B**) Starting pH 7 at 40 °C. (**C**) Starting pH 8 at 40 °C. (**D**) Starting pH 9.5 at 40 °C. Each condition was repeated three times. Additional stability data for these trials are available in Figure S1. All 1% solutions are in blue; 0.5% in green; and 0.25% in black. A single-factor ANOVA was used to test for differences in starting pH values and for differences in concentrations.

The stability of NCT was tested at three concentrations (1%, 0.5%, and 0.25%) at starting unbuffered pH adjusted values of 7, 8, and 9.5, both at ambient temperature and 40 °C. The stability of the NCT solutions was monitored by UV-Vis (Figure 3). Table 1 shows the half-life values calculated for each pH value and temperature. There was no significant difference in the half-life values for the various initial starting pH values at the tested temperatures. Half-life values were concentration-dependent (*p* < 0.05) and increased from ~15 days for 1% NCT solutions to ~45 days for 0.25% solutions at 40 °C. More variation was seen in the room temperature solutions; however, the half-life values increased from ~121 days for 1% NCT solutions to nearly double (~237 days) with 0.5% NCT solutions. At 0.25%, the variability was too large to allow for precise half-life determination because the rate of decay was so slow; longer testing periods would be required for a more reliable measurement of this characteristic. 

**Figure 4:**
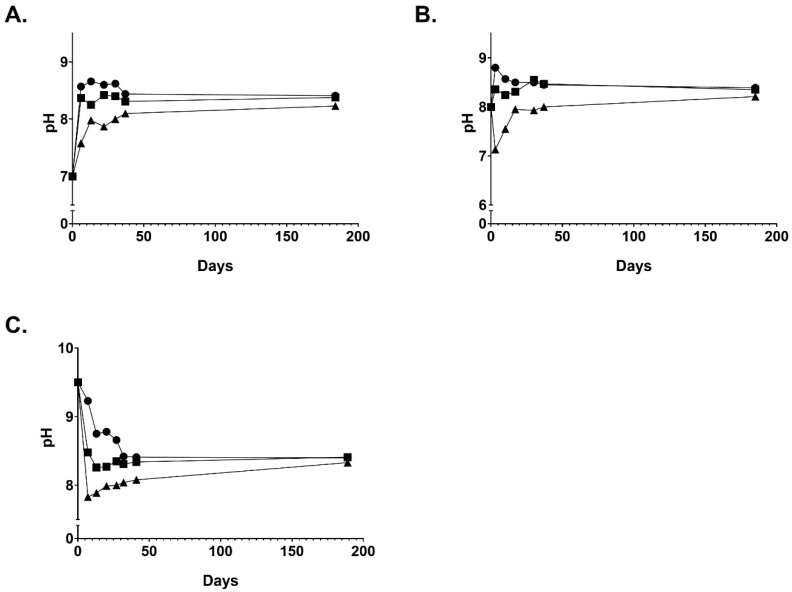
pH of aqueous NCT solutions at 1% (circle), 0.5% (square), and 0.25% (triangle). (**A**) Solutions starting at a pH value of 7. (**B**) Solutions starting at a pH value of 8. (**C**) Solutions starting at a pH value of 9.5. Additional pH data for the three trials are available in Figure S2.

The pH of these solutions was monitored over this same time period and converged at a value of ~8.3 (Figure 4 and Figure S2). The pH 7 solutions increased in pH in the testing period while solutions starting at pH 9.5 decreased. A similar trend was seen with samples tested at 40 °C (Figure S2). 

**Figure 5:**
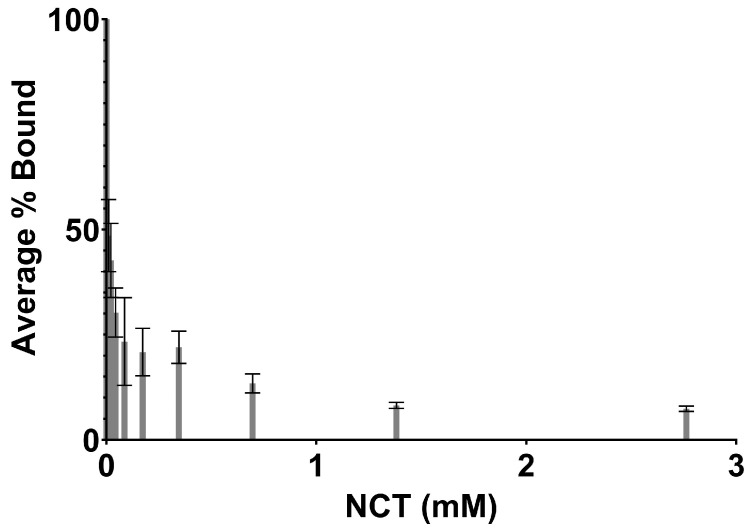
ELISA test results for IL-6 binding to an IL-6 specific antibody after treatment with concentrations of NCT ranging from 0 to 2.76 mM. A single-factor ANOVA was used to test for differences in binding at all concentrations of NCT tested.

The capacity of NCT to modify IL-6 cytokine was tested with the fully characterized compound at various concentrations. As seen in Figure 5, the binding of IL-6 to an IL-6-specific antibody decreased by ~93% after treatment with 2.76 mM NCT. All concentrations of NCT tested resulted in IL-6 binding that was significantly different from the control (*p* < 0.05).

**Figure 6:**
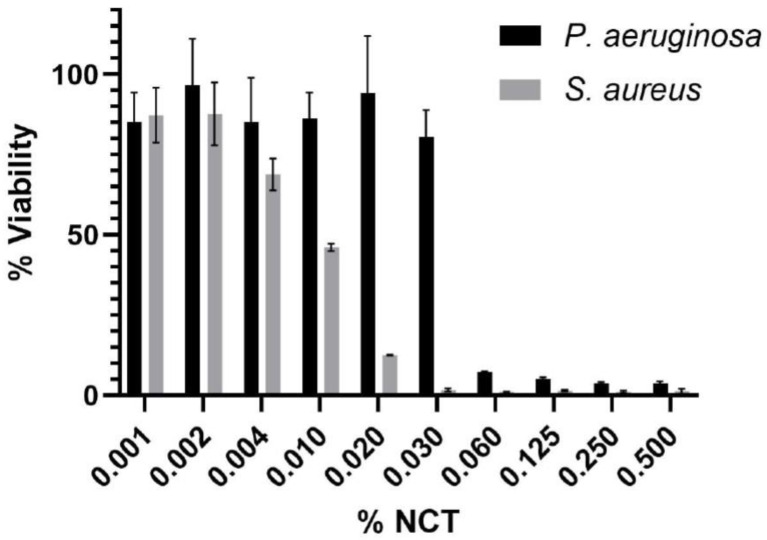
Viability of remaining *P. aeruginosa* (black) and *S. aureus* (gray) biofilm biomass after treatment with various NCT concentrations. Significant reductions in viability were calculated using ANOVA and Tukey’s post hoc analysis. The significant threshold concentrations were 0.004% for *S. aureus* and 0.06% for *P. aeruginosa*.

Antimicrobial efficacy testing of NCT versus *Pseudomonas aeruginosa* and *Staphylococcus aureus* cultures resulted in MICs of 125 µg/mL for both organisms. The MBEC values for *P. aeruginosa* and *S. aureus* were 500 µg/mL and 250 µg /mL, respectively. Additionally, biofilm reduction and Cell Titre Blue (Promega UK) assays were conducted to determine if NCT impacts the biomass and viability of established (mature) biofilms. No significant reduction in biofilm biomass was seen for *P. aeruginosa* or *S. aureus* 24 h post-treatment (Figure S3). However, the viability of the remaining biomass was reduced by ~87% and ~93% at NCT concentrations of 2000 µg /mL and 6000 µg /mL for *S. aureus* and *P. aeruginosa*, respectively (Figure 6). 

## 3. Discussion

Our analytical data obtained by UV-Vis spectroscopy, IR spectroscopy, NMR spectrometry, and HPLC confirm the successful synthesis of NCT in a highly pure form by the method of Gottardi and Nagl [3]. Previous studies applied electrospray ionization mass spectrometry to the analysis of NCT, giving an M^+^ and MH^+^ of 180.1 and 181.1 g/mol [3,7]. Here, we used LCMS to verify the exact mass of NCT with a single peak at 252 nm and purity of >95% (Figure 2). To the best of our knowledge, this is the first time the elimination product has been detected in NCT solutions. The elimination product has been proposed as an intermediate prior to the formation of sulphoacetaldehyde in vivo. The first-order rate constant for sulphoacetaldehyde formation from NCT at 37 °C and pH 7.4 is 9.9 × 10^−4^·h^−1^ [8].

Previously reported studies on the stability of 0.75% NCT solutions at room temperature and at 2–5 °C showed declines in titratable active chlorine (Cl^+1^) of 0.43%/day and 0.03%/day, respectively [3]. Our results on the long-term trends of stability of aqueous NCT solutions at various concentrations, temperatures, and initial pH values expand on those reported by Gottardi and Nagl [3]. The extended Cl^+1^ half-lives we observed at room and elevated temperatures show how well-suited solutions of NCT are for practical applications in healthcare. The pH shifts that we saw in all solutions were rapid, and clearly differed by concentration in the early stages but leveled off after 14 days until each arrived at a constant pH value near 8.3. These findings extend our understanding of the behavior of NCT in water, which was previously limited to observations of pH shifts in the first 12 h after preparation of 5.85–54.6 mM (0.1–1%) aqueous solutions of NCT, before subsequently reaching a constant pH level [3].

Thorough antimicrobial analyses allowed us to reveal additional characteristics of NCT. Previous studies on planktonic growths of *S. aureus* and *P. aeruginosa* indicate >5 log reduction values for both bacteria in the presence of 1% NCT [2]. MIC concentrations (125 µg/mL, 0.69 mM) determined in this study confirm and expand on published observations by showing that low concentrations of NCT are effective not only against *P. aeruginosa*, but *S. aureus* as well [9].

Our study has also enabled quantitative evaluation of anti-biofilm efficacy. Successful biofilm treatments must be able to prevent bacterial attachment and disrupt or kill established biofilms. Concentrations of NCT (300 µM, 0.005%) were reported to be ineffective at killing and preventing biofilm growth for established biofilms of *P. aeruginosa* [9]. Our MBEC results confirm this finding and also show that mM concentrations of NCT are required for the inhibition of biofilms for both *S. aureus* and *P. aeruginosa*. It has also been reported that 48 h established biofilms of *S. aureus* and *P. aeruginosa* on metal coupons can be reduced by >4 log reduction values in 1 h and 30 min, respectively, with 1% NCT. SEM images in this same study also indicate that 1% NCT disrupts the *S. aureus* biofilm matrix [10]. In contrast, we observed that 24 h established biofilm on polystyrene is not disrupted by 1% NCT, which is in keeping with what is understood about the recalcitrance of established biofilms to topical biocides [11]. However, despite this, we observed that the remaining biomass was largely non-viable, confirming that NCT can kill bacteria in an established biofilm. That ability is crucial if NCT is to be taken forward as a topical treatment for chronic, biofilm-associated infections.

The mechanism of NCT reactivity can be impacted by the experimental system. For example, NCT can react directly with biological materials including amines, thiols, biological fluids, and culture media. Some of these direct modifications quench the active chlorine (e.g., oxidation of thiols) while others shuttle the active chlorine for further use (e.g., amines) and have the potential to increase or decrease the activity of NCT. This may be true for potential product formulation components as well. In this study, we used static culture systems and nutrient broth, which contains high concentrations of proteins. Despite these conditions, NCT had the capacity to inhibit bacterial growth and kill biofilm organisms.

Complementary to the antibacterial and anti-biofilm activity is our observation that IL-6 binding is also impaired by exposure to NCT, an effect that could have therapeutic or prophylactic utility in the management of inflammation in vivo. The concentration of NCT necessary to reduce IL-6 binding by 50% is ~5 times that of HOCl, a well-established modulator of IL-6 [5]. Wound chronicity, for example, is maintained in part by an aberrant immune response creating a pro-inflammatory state in the lesion. Treatment of autoimmune diseases such as rheumatoid arthritis conventionally relies on anti-IL-6 antibodies [12]. NCT offers potential not only as a topically applied agent for wound healing but also as an alternative therapeutic approach to systemic inflammatory diseases. 

NCT preparations that are demonstrably stable, pure, efficacious, and inexpensive to manufacture have the potential to provide an accessible way to manage a variety of clinical conditions in both developed and developing countries. Effective concentrations appear to be low enough for NCT to find use in the management of infected wounds. Given the current global burden and cost of chronic wound management, these attributes position NCT as an attractive option for continued development. 

## 4. Materials and Methods

Chloramine-T trihydrate 98%, taurine 99%, ethyl alcohol 99.5+%, ammonium acetate, and acetonitrile (HPLC grade) were purchased from Thermo Fisher Scientific (Waltham, MA, USA). Water was obtained from a Milli-Q water purification system. D_2_O 99.9% was purchased from Cambridge Isotope Laboratories Inc. (Tewksbury, MA, USA). 

Synthesis of NCT. NCT was synthesized as crystalline sodium salt following the procedures in [3]. 

Preparation of Aqueous NCT Samples. Samples consisting of 1 L of 1%, 0.5%, and 0.25% NCT were made in water with initial pH values of 7, 8, and 9.5. The pH of the water was adjusted with 0.1 M NaOH or HCl. The samples were aliquoted into 100 mL amber glass bottles. Room temperature samples were kept in a closed cabinet. All samples were kept out of direct sunlight. Elevated temperature samples were placed in an incubator at 40 °C. A fresh aliquot was used for analysis. The pH of the samples was monitored with an Accumet pH meter from Fisher Scientific. Three samples were made at each concentration and temperature. 

UV-Vis Spectrophotometry. Solutions of NCT were made in milli-Q water; N,N-dichlorotaurine was made in 0.1 M HCl. The spectrophotometer was blanked with Milli-Q prior to use and wavelength scans (200–400 nm) were collected using a Thermo-Scientific BioMate 3S. All samples were measured at 252 nm and diluted to fall within the linear range. The extinction coefficient used for NCT was 395.5 M^−1^·cm^−1^ [3].

IR Spectroscopy. Solid samples of NCT and taurine were analyzed (8 scans) using Nicolet iS50 FTIR with ATR from Thermo-Scientific. 

NMR Spectroscopy. Samples were prepared by dissolving 10 mg of NCT or taurine into 750 μL of D_2_O. ^13^C data were collected (1024 scans) using a Bruker 400 MHz NMR spectrophotometer (Bruker, Billerica, MA, USA). 

Liquid chromatography–mass spectroscopy (LCMS). A solution of NCT (1 mg/mL in Milli-Q water) was prepared. Each sample (3.00 μL) was analyzed by LCMS. An Agilent 1290 Infinity UHPLC system (Agilent, Santa Clara, CA, USA) coupled to an Agilent 6230B time-of-flight mass spectrometer (TOF-MS) (Agilent) equipped with a dual Agilent Jet Stream electrospray ionization source (Agilent) was used for compound separation and analysis. The separation was performed with a ZORBAX RRHD Eclipse Plus C18 column (50 mm × 2.1 mm i.d., 95 Å, 1.8 μm) using 50% (*v*/*v*) 50/50 of 50 mM ammonium acetate (pH = 6.8) and acetonitrile at a 300 μL/min flow rate for 4 min and a column temperature of 20 °C.

The TOF-MS instrument was operated in negative ionization mode with the following settings: 325 °C drying gas temperature with an 8 L/min flow rate, 350 °C sheath gas temperature with an 11 L/min flow rate, and 30 psig nebulizer pressure. Voltage parameters were set as follows: 3500 V capillary, 65 V skimmer, 1000 V nozzle, and 175 V fragmentor. 

The data acquisition was performed in the standard mass range (≤*m*/*z* 3200) with 2 GHz extended dynamic range mode at an acquisition rate of 1.0 spectra/s. Data were analyzed using Agilent Qualitative Analysis software (v 10.0). Spectral peak data were background subtracted using pre-injection data.

Titration of NCT. Iodometric titrations of NCT solutions using sodium thiosulfate (0.113 N) were completed following the HACH method 8209.

IL-6 ELISA. Following our previous methods, the Invitrogen human IL-6 ELISA kit (catalog # 88-7066, Invitrogen, Waltham, MA, USA) was purchased and used according to the provided protocol [5]. Briefly, 96-well plates were coated with the capture antibody (anti-human-IL-6 antibody). IL-6 (150–200 pg/mL in water) was treated with various concentrations of NCT (0–2.76 mM) for 5 min at room temperature and then quenched with 0.05% sodium thiosulfate (STS). The exact concentrations tested were 0, 2.76 mM, 1.38 mM, 0.69 mM, 0.35 mM, 0.17 mM, 0.086 mM, 0.043 mM, 0.022 mM, and 0.011 mM. The antibody for human IL-6 conjugated with HRP was used to detect the antigen. All experiments were conducted a minimum of three times. Samples for each experiment were plated in triplicate. The graph includes averages and standard deviations. A single-factor ANOVA was used to test for differences in binding at all concentrations of NCT tested. 

Bacterial strains and culture conditions. *Pseudomonas aeruginosa* ATCC9027 and *Staphylococcus aureus* EMRSA-15 (NCTC 13616) were routinely cultured on nutrient agar (NA; Sigma Aldrich, St. Louis, MI, USA) or in nutrient broth (NB; Sigma Aldrich) at 37 °C. Both strains are originally of skin origin. All experiments were conducted a minimum of three times in triplicate.

Minimum Inhibitory Concentration (MIC). MICs used the broth microdilution method, as recommended by the Clinical and Laboratory Standards Institute [13]. An overnight inoculum was freshly adjusted in Mueller–Hinton Broth (Sigma Aldrich) to McFarland 0.5 turbidity standard. An equal volume of adjusted bacterial suspension and a range of final concentration 0.001–0.5% (*w*/*v*) NCT were added to each of the wells of the plate (final concentration of Mueller–Hinton Broth was 50%). The plates were incubated for 16 h at 37 °C. The negative controls were media only and the positive controls were untreated. Turbidity was measured at A_620_ (SPECTROstar Nano; BMG Labtech, Ortenberg, Germany); the MIC was taken as the lowest concentration where no growth was observed.

Minimum Biofilm Exclusion Concentration (MBEC). MBEC is defined as the lowest concentration of an antimicrobial agent required to inhibit biofilm growth [14]. Pre-cultures of each bacterium were freshly seeded into a 96-well microtitre plate in NB (Sigma Aldrich) at a cell density of 1 × 10^6^ CFU/mL. A range of final concentrations of 0.001–0.5% (*w*/*v*) NCT were added to each of the wells. The plates were incubated at 37 °C for 24 h. The wells were washed three times with sterile phosphate-buffered saline (PBS; Sigma Aldrich) to remove non-adherent cells and then stained with 0.1% crystal violet (Sigma-Aldrich) for 5 min. The wells were washed three times with sterile PBS and destained with 7% (*v*/*v*) acetic acid. The plates were read at A_595_ (SPECTROstar Nano; BMG Labtech). The negative controls were media only and the positive controls were untreated. 

Biofilm disruption and viability. Pre-cultures of each bacterium were freshly seeded into a 96-well microtiter plate in nutrient broth (Sigma Aldrich) at a cell density of 1 × 10^6^ CFU/mL and incubated for 24 h at 37 °C. Media and planktonic organisms were aspirated and discarded. Then, the wells were washed three times with sterile PBS water to remove loosely adherent cells. The fresh nutrient broth was added to each well in the final concentration range of 0.001–0.5% (*w*/*v*) NCT. The plates were incubated for a further 24 h.

To measure the remaining biofilm biomass, media was aspirated from each well and discarded, and the wells were washed three times with sterile PBS. Biomass was stained with crystal violet as described for MBEC. The viability of the biofilm biomass was established using Cell Titre Blue (Promega, Madison, WI, USA), according to the manufacturer’s instructions. Media was aspirated from each well and discarded, and the wells were washed three times with sterile PBS. Then, 200 μl of Cell Titre Blue reagent was added to each well and incubated in the dark, at room temperature for 1 h. Viability was measured using a Tecan Infinite^®^ (Tecan, Männedorf, Switzerland) plate reader (excitation: 550 nm; emission 600 nm) and calculated as a percentage change compared to the untreated control. Significant reductions in biofilm biomass and viability were calculated using ANOVA and Tukey’s post hoc analysis. 

Statistical Analysis. A single-factor ANOVA was used to test for differences in the half-lives for starting pH values and for differences in concentrations. A single-factor ANOVA was used to test for differences in binding at all concentrations of NCT tested. Significant reductions in biofilm biomass and viability were calculated using ANOVA and Tukey’s post hoc analysis.

## Figures and Tables

**Table 1:** Average half-life values for three different concentrations of NCT and starting pH values at room temperature and 40 °C. Each experiment was repeated three times. A single-factor ANOVA was used to test for differences in half-life at various pH values.

		Half-Life (Days)		
		pH 7	pH 8	pH 9.5
**RT**	1%	121.5 ± 14.9	124.5 ± 8.0	116.6 ±18.1
	0.50%	222.7 ± 40.5	238.4 ± 28.4	248.7 ± 77.8
	0.25%	* 539.0 ± 307.1	* 345.6 ± 238.6	* 289.0 ± 75.8
**40 °C**	1%	14.5 ± 0.5	15.0 ± 1.0	15.9 ± 1.6
	0.50%	26.3 ± 3.2	26.6 ± 3.4	26.3 ± 3.2
	0.25%	41.8 ± 6.1	43.4 ± 3.1	48.5 ± 8.6

* Large variability does not allow for precise half-life determination.

## Data Availability

Data sharing is not applicable to this article.

## Supplementary Materials

The following supporting information can be downloaded at https://www.mdpi.com/article/10.3390/ijms25168745/s1.
