# Supplementary material for: Long-Term Stability and Efficacy of NCT Solutions
Source: Int J Mol Sci. 2024 Aug 10;25(16):8745. doi: 10.3390/ijms25168745 (PMC11354306; doi:10.3390/ijms25168745)
Supplement: Supplementary file 1 [file ijms-25-08745-s001.zip › ijms-3062452-supplementary.pdf]

Long-term stability and efficacy of NCT solutions

Gabriel J. Staudinger [1†], Zach M. Thomas [1†], Sarah E. Hooper [2], Jeffrey F. Williams [3] and Lori I. Robins [1*]*

1 Physical Sciences Division, University of Washington Bothell, Bothell, Washington, United States of America.
2 Microbiology & Infection Research Group, Cardiff School of Sport and Health Sciences, Cardiff Metropolitan University, Cardiff, CF5 2YB, United Kingdom
3 Briotech Inc. 1102 Shuksan Way, Everett, WA 98203
† These authors contributed equally to the manuscript
* Correspondence: lrobins@uw.edu

Table of Contents

Figure S1. Stability of aqueous NCT solutions at various concentrations and starting pH values. **S3**

Figure S2. pH of aqueous NCT solutions at 1%, 0.5%, and 0.25% starting at pH values of 9.5, 8, and 7. .... **S4**

Figure S3. Removal of remaining *P. aeruginosa* (blue) and *S. aureus* (orange) biofilm biomass after treatment with various NCT concentrations. **S5**

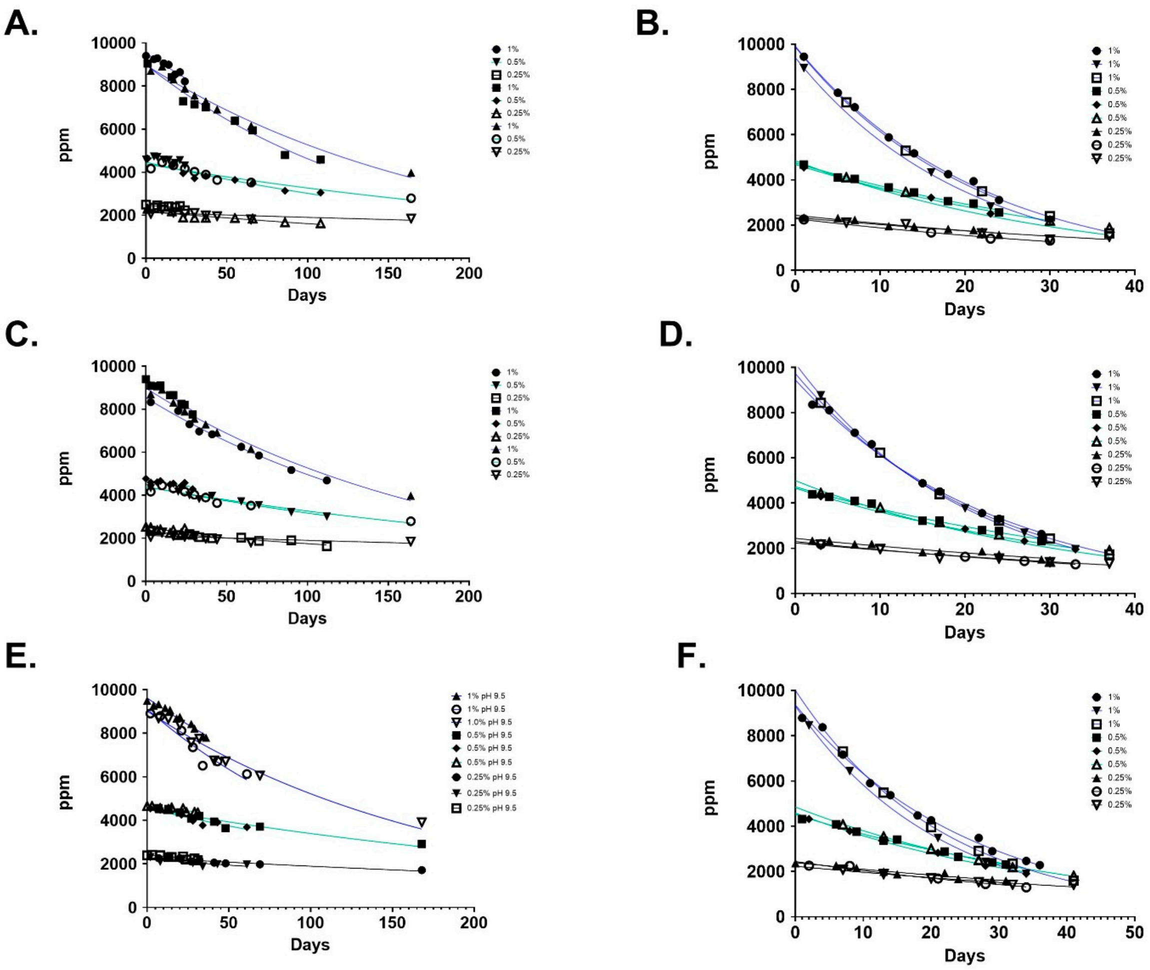

Figure S1. Stability of aqueous NCT solutions at various concentrations and starting pH values. NCT solutions starting at pH 7 at 1%, 0.5%, and 0.25% NCT at room temperature (A) and 40 °C (B). NCT solutions starting at pH 8 at 1%, 0.5%, and 0.25% NCT at room temperature (C) and 40 °C (D). NCT solutions starting at pH 9.5 at 1%, 0.5%, and 0.25% NCT at room temperature (E) and 40 °C (F). A single factor ANOVA was used to test for differences in starting pH values and for differences in concentrations. All 1% NCT trials are in blue; 0.5% NCT trials are in green; 0.25% NCT trials are black.

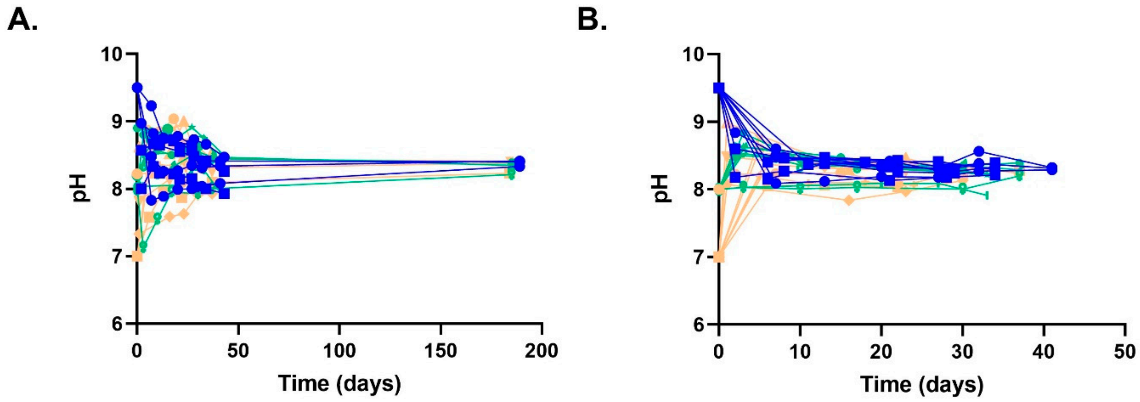

Figure S2. pH of aqueous NCT solutions at 1%, 0.5%, and 0.25% starting at pH values of 9.5, 8, and 7 at room temperature (**A**) and 40 °C (**B**). Trials at pH 9.5 are blue; trials at pH 8 are green; trials at pH 7 are orange.

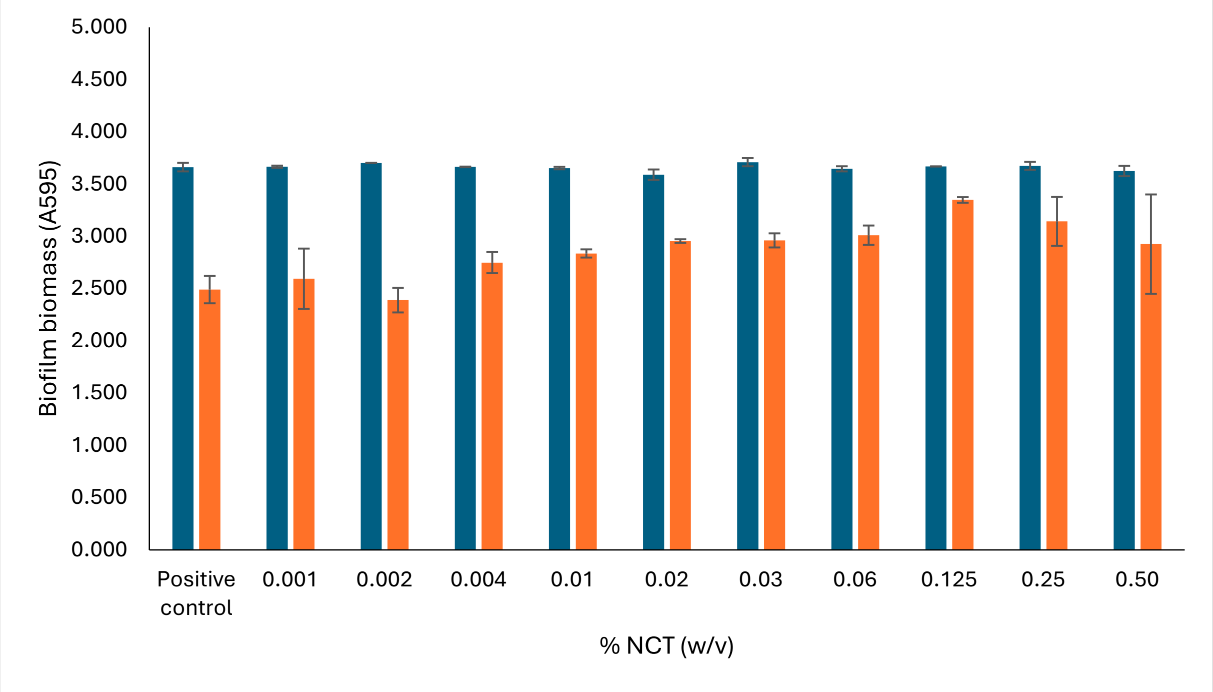

Figure S3. Removal of remaining *P. aeruginosa* (blue) and *S. aureus* (orange) biofilm biomass after treatment with various NCT concentrations. Significant reductions in biofilm biomass were calculated using a single factor ANOVA ($p>0.05$).
